# Draft Genome Sequence of *Photorhabdus luminescens* subsp. *laumondii* HP88, an Entomopathogenic Bacterium Isolated from Nematodes

**DOI:** 10.1128/genomeA.00154-16

**Published:** 2016-03-17

**Authors:** Shimaa Ghazal, Rediet Oshone, Stephen Simpson, Krystalynne Morris, Feseha Abebe-Akele, W. Kelley Thomas, Kamal M. Khalil, Louis S. Tisa

**Affiliations:** a University of New Hampshire, Durham, New Hampshire, USA; b Genetic Engineering & Biotechnology Division, Genetics and Cytology Department, Applied Microbial Genetics Laboratory, National Research Centre, Dokki, Cairo, Egypt

## Abstract

*Photorhabdus luminescens* subsp. *laumondii* HP88 is an entomopathogenic bacterium that forms a symbiotic association with *Heterorhabditis* nematodes. We report here a 5.27-Mbp draft genome sequence for *P. luminescens* subsp. *laumondii* HP88, with a G+C content of 42.4% and containing 4,243 candidate protein-coding genes.

## GENOME ANNOUNCEMENT

*Photorhabdus* species are Gram-negative motile bioluminescent bacteria that maintain two distinct lifestyles as insect pathogens and in a symbiotic relationship with the entomopathogenic *Heterorhabditis* nematodes (see references 1–7 for a review). The life cycles of *Photorhabdus* and its nematode host *Heterorhabditis* are best described as a cyclic association that begins and ends with infective juvenile (IJ) nematodes. A monoculture of *Photorhabdus* is maintained within the anterior region of the IJ nematode’s intestine (8, 9). The nematodes actively seek and infect insect hosts by entering through natural openings or by burrowing directly through the insect cuticle. Once inside the insect, the nematodes regurgitate the bacteria into the hemolymph (8). The bacteria release highly virulent toxins (10, 11), which results in insect death in <48 h. As the bacteria enter the stationary phase of their growth cycle, they secrete extracellular enzymes that aid in breaking down insect tissue, thereby providing nutrients for both the bacteria and nematodes. The bacteria also generate essential growth factors for the nematode growth and development. The growth and development of *Heterorhabditis* nematodes have an obligate requirement for their specific bacterial symbiont (12). The bacteria also release antibiotics to prevent secondary invaders and putrefaction of the insect carcass (13, 14). After several days of feeding, the nematodes and bacteria reassociate and leave in search of a new insect host.

Based on molecular analysis, the *Photorhabdus* genus is divided into three bacterial species: *P. luminescens*, *P. temperata*, and *P. asymbiotica* (15, 16). Our understanding of these bacteria has been greatly enhanced by the genome sequencing of strains from all three established species: *P. luminescens* TT01 (17), *P. asymbiotica* ATCC 43949 (18, 19), *P. temperata* NC19 (20), *P. temperata* Meg1 (21), *P. luminescens* BA1 (22), *P. asymbiotica* Kingcliff (23), and *P. temperata* M121 (24). Here, we present a draft genome sequence for *P. luminescens* subsp. *laumondii* HP88, which was isolated from *Heterorhabditis bacteriophora* nematodes found in Utah (25).

The draft genome of *P. luminescens* strain HP88 was generated at the Hubbard Genome Center (University of New Hampshire, Durham, NH) using Illumina technology (26) techniques. A standard Illumina shotgun library was constructed and sequenced using the Illumina HiSeq 2000 platform, which generated 7,680,248 reads (260-bp insert size) totaling 1,120.0 Mbp. The Illumina sequence data were assembled using CLC Genomics Workbench (version 8.5) and AllPaths-LG (version r41043) (27). The final draft assembly contained 287 contigs, with an *N*_50_ of 34.4 kb. The total size of the genome is 5.27 Mbp, and the final assembly is based on 949 Mb of Illumina draft data, providing an average 163× coverage of the genome.

The high-quality draft genome of *P. luminescens* strain HP99 was resolved to 287 contigs consisting of 5,268,230 bp, with a G+C content of 42.4%. The assembled *P. luminescens* strain HP88 genome was annotated via the NCBI Prokaryotic Genome Annotation Pipeline (PGAP) and resulted in 4,243 candidate protein-coding genes.

### Nucleotide sequence accession numbers.

This whole-genome shotgun project has been deposited at DDBJ/EMBL/GenBank under the accession no. LJPB00000000. The version described in this paper is version LJPB01000000.
